# Implant-Supported Prostheses in the Edentulous Mandible: Biomechanical Analysis of Different Implant Configurations via Finite Element Analysis

**DOI:** 10.3390/dj11010004

**Published:** 2022-12-23

**Authors:** Eduardo Anitua, Naiara Larrazabal Saez de Ibarra, Luis Saracho Rotaeche

**Affiliations:** BTI Biotechnology Institute, Jacinto Quincoces 39, 01007 Vitoria, Spain

**Keywords:** biomechanics, edentulous mandible, dental implant, finite element analysis

## Abstract

This study explores the implant-supported prosthetic treatment alternatives of the edentulous mandible from a biomechanical point of view by means of a Finite Element Analysis (FEA). Finite element (FE) models were used to simulate cases treated with six, five, and four, implants and a fixed prosthesis with a cantilever. In the four implant treatments, three cases were analyzed; the posterior implants were placed in axial positions, angled at 30° and 45°. Cases with six and four axially placed implants were also analyzed by placing the posterior implants distally to the foramen, thus eliminating the cantilever in the prostheses. In the cases with implants between foramina, the highest values for the principal strains and von Mises stresses were observed in the case with four implants where the posterior implants were angled at 45°. Cases with implants placed distally to the foramen and without a cantilever showed much lower bone stress and strain levels compared to cases with implants between foramina. From a biomechanical point of view, it seems to be a better option to use implants positioned distally to the foramen, eliminating cantilevers.

## 1. Introduction

Implant-supported prosthetic treatments with implants placed between the foramina were first described by Adell et al. [1] in the 1980s, who presented a 15-year study of 410 edentulous mandibles in which 5 or 6 implants were placed between foramina. To facilitate the treatment in patients without sufficient bone volume, techniques such as bone grafts, guided bone regeneration, and distraction osteogenesis were developed to allow the insertion of standard-length implants [2,3]. In order to prevent the complications of these treatments and their high costs, Maló et al. [4] presented an alternative for the treatment of edentulous mandibles that claimed to be simpler and cost-saving, which was based on placing only four implants (the all-on-four concept). In the all-on-four treatment, the four implants are placed between foramina, with the two posterior implants angled at approximately 30° in order to reduce the distal cantilever of the prosthesis [5,6]. A variety of posterior surgical configurations that differ from the all-on-four original concept for the treatment of edentulous mandibles can be found in the literature, where the use of two to six (or more) implants has been suggested [7]. The development of short and extra-short implants represented new alternatives to avoid the need for additional surgeries for bone augmentation and the angulation of implants [8,9,10]. By using these types of implants, it is possible in many cases to place the posterior implants distal to the foramen, thus eliminating or considerably reducing the cantilever of the prosthesis. Moreover, the need for complex additional surgical techniques to house the implants is reduced when the available bone by anatomical structures, such as the dental nerve, is limited [9,11]. Nevertheless, it should be mentioned that implant length is a controversial topic, with some authors reporting lower success rates for short and extra short implants [12,13,14,15,16], whereas others report very high survival rates [17,18,19,20]. These discrepancies may be explained by the fact that short and extra short implants are mainly used in complicated clinical situations, where the experiences and skills of the clinicians are critical. In any case, the effect of the implant length has been reported to be much lower than that of other parameters, such as the implant’s body diameter [21,22,23]. In fact, in the implant–bone interface, where excessive strain may lead to bone loss, stress is mainly distributed along the first six threads of the implant [24], with the peak stress being at the bone crest level [25,26,27]. Consequently, unnecessarily increasing the length of the implant may result in limited improvements, even though a longer implant may improve primary stability in situations where the cancellous bone is predominant [26,28].

From a biomechanical point of view, and to achieve a better understanding of the benefits of different implant treatment alternatives for edentulous mandibles, several studies have used the finite element analysis (FEA) [29,30,31,32,33,34,35,36,37] to analyze the stresses induced at the implant–bone interface, which have been shown to have significant effects on biological bone resorption processes that can lead to implant failures [38]. Regarding the framework material, Kelkar et al. concluded that the framework material has an influence on the stress distribution pattern at the implant–bone interface [39]. The study performed by Dayan et al. follows the same direction, adding that stiff materials reduce stress in peri-implant bones when the distal implants are tilted 30° [40]. Other authors also stated that stiffer framework materials reduce the forces and, consequently, the stresses, supported by the abutment [41] and the prosthetic screw [42]. Concerning the implant’s configuration, several studies analyzed the all-on-four technique. There is a consensus that implant tilting generates an increase in peri-implant stresses. Some authors recommend implant tilting to reduce cantilevers [42], thus reducing the stresses at the implant–bone interface [43,44].

This study aims to extend the knowledge obtained from the aforementioned research by adding the alternative of the cantilever reduction or elimination by means of the axial placement of short or extra-short implants. This technique may not only reduce the stress by eliminating the cantilever but also by placing the implants axially, thus presumably reducing the stresses at the implant–bone interface. For this purpose, the present study carries out a biomechanical analysis simulating various cases with six, five, and four implants and studying the effect of eliminating the cantilever by means of short implants. In this sense, the hypothesis of this study is that cantilever reduction by using short implants provides better results than tilted implants (all-on-four) in terms of strains and stresses transmitted to the bone.

## 2. Materials and Methods

**Figure 1:**
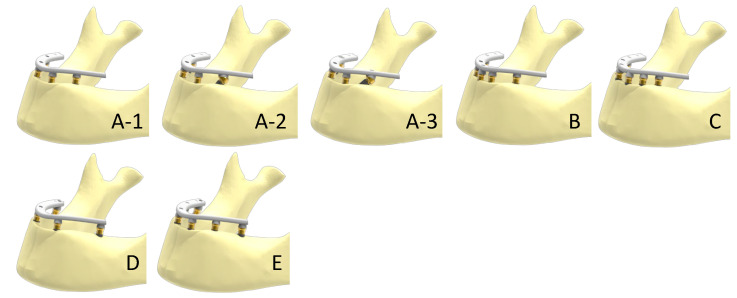
Above—scenarios with all implants between foramina. A-1: 4 implants. A-2: 4 implants, with posterior implants tilted 30°, keeping the fulcrum shaft at the same point as in case A-1. A-3: 4 implants, with posterior implants tilted 45°, keeping the fulcrum shaft at the same point as in case A-1. B: 5 axial implants. C: 6 axial implants. Below—cases with posterior implants placed distally to the foramina, avoiding the cantilever. D: 4 axial implants. E: 6 axial implants. In A (A-1, A-2, and A-3), B, and C scenarios, the cantilever lengths were unchanged (11.5 mm) by keeping the emergence points of the posterior implants fixed. In scenarios D and E, the cantilever was eliminated by placing the posterior implants distally to the foramina.

**Figure 2:**
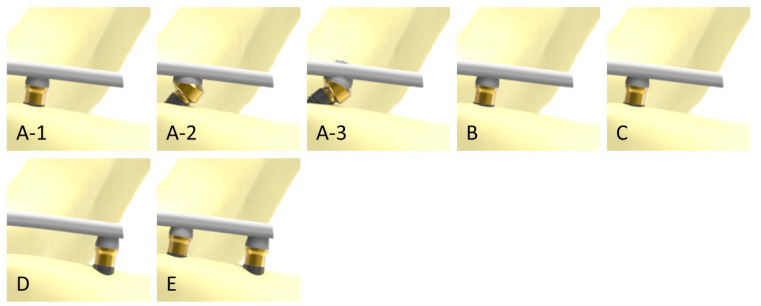
Above—details of the cantilevers in scenarios A-1, A-2, A-3, B, and C. Below—scenarios D and E without cantilevers.

To generate finite element (FE) models, a three-dimensional geometry of an edentulous mandible was used as the starting point, which was in turn divided into two volumes representing the trabecular and cortical bones. Based on this, different scenarios were generated using the CAD design software Solidworks 2020^®^ (Dassault Systèmes, Vélizy-Villacoublay, France). The number and configuration of implants in each simulation are described in Table 1 while their corresponding FE models are shown in Figure 1 and the details of the cantilevers are shown in Figure 2.

The 7.5 mm-length implants (CORE^®^, BTI Biotechnology Institute, Vitoria, Spain) were used in all scenarios with Ø3.5 mm in the anterior regions and Ø3.75 mm in the posterior regions (both with the same Ø3.5 mm platform). Axially placed implants mounted 3 mm-high straight transepithelial abutments (intermediate abutments) (Multi-Im^®^, BTI Biotechnology Institute, Vitoria, Spain) with a Ø3.5 mm implant platform and Ø4.1 mm prosthetic platform. Moreover, tilted implants mounted 30° and 45°-angled transepithelial abutments with a Ø3.5 mm implant platform and Ø5 mm prosthetic platform. Screw-retained CoCr alloyed full-arch fixed prostheses (6 mm high from the implant platforms) were used in all scenarios. The implant materials, all prosthetic components, as well as their mechanical properties are described in Table 2.

**Figure 3:**
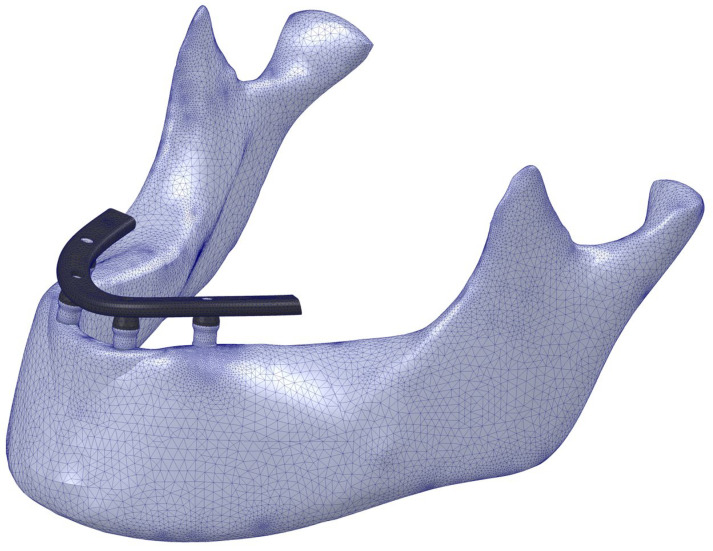
FE model mesh of case 4-A, the mesh refinement area in the ROI can be observed.

Solidworks Simulation Premium 2020^®^ software (Dassault Systèmes, Vélizy-Villacoublay, France) was used to generate the FE models. The models were meshed by using 10-node tetrahedral elements with maximum element sizes of 1.5 mm and minimum element sizes of 0.25 mm. In the region of interest (ROI) of the model, a sub-model was performed to refine the mesh in this area, using an element size of 0.25 mm (Figure 3) following the recommendations provided by Sato et al., who concluded that an element size of 0.3 mm was adequate for modeling the bone-implant interface [45].

**Figure 4:**
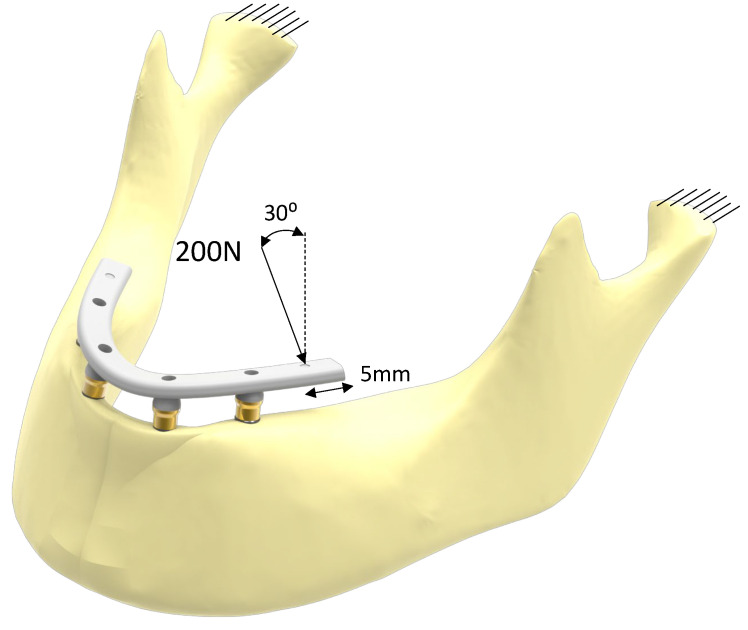
Boundary conditions and application of the load on the model.

All materials were modeled as homogeneous, isotropic, and linear. The material properties of each of the components are described in Table 2. The mesh between the implant interface and the bone tissue was performed by means of a rigid bond contact with compatible mesh (the entities in contact were meshed in such a way that there was a node-to-node correspondence between the meshes of each entity), simulating a stage of complete bone-to-implant osseointegration. For the remaining connections among components, a rigid bonding contact was also used, although in this case, the mesh was not compatible between the contact surfaces. All degrees of freedom were restricted at the articular face of the condyles and a load of 200 N angled at 30° was applied at a distance of 5 mm from the distal end of the prosthesis (Figure 4) [32].

**Table 2:** Material and mechanical properties of the components.

Component	Material	Elastic Modulus (MPa)	Poisson Coef.
Dental implant	Pure titanium [46]	105,000	0.37
Superstructure retaining screw	Titanium alloy [46]	113,800	0.342
Transepithelial body	Pure titanium [46]	105,000	0.37
Transepithelial screw	Titanium alloy [46]	113,800	0.342
Prostheses	CrCo alloy [47]	218,000	0.33
Bone	Cortical bone [47]	13,700	0.28
	Trabecular bone [47]	1370	0.3

## 3. Results

Maximum and minimum principal strains and the equivalent von Mises stresses in the ROIs were recorded in each scenario, only considering the stresses and strains in the bone tissue. Table 3 shows the maximum value of the max. principal strain, the minimum value of the min. principal strain, and the maximum value of the von Mises stress in the bone for each scenario.

**Figure 5:**
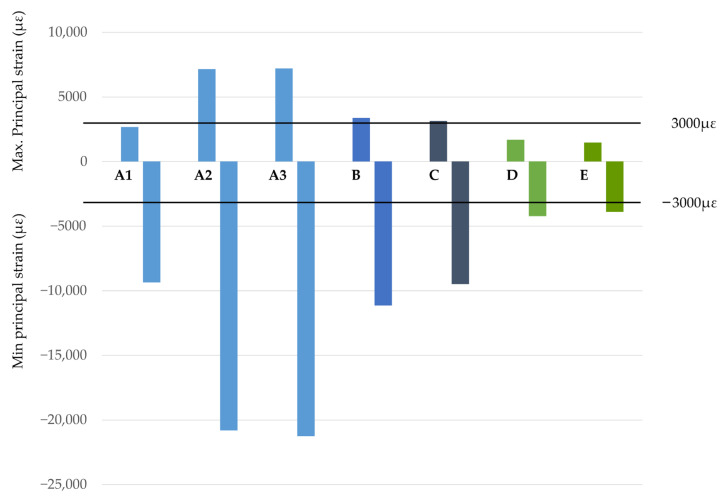
Maximum values for the max. principal strain (µε) and minimum values for the min. principal strain (µε). Blue shades—cases with implants between foramina. Green shades—cases with the 2 implants posterior to the distal of the foramen.

**Figure 6:**
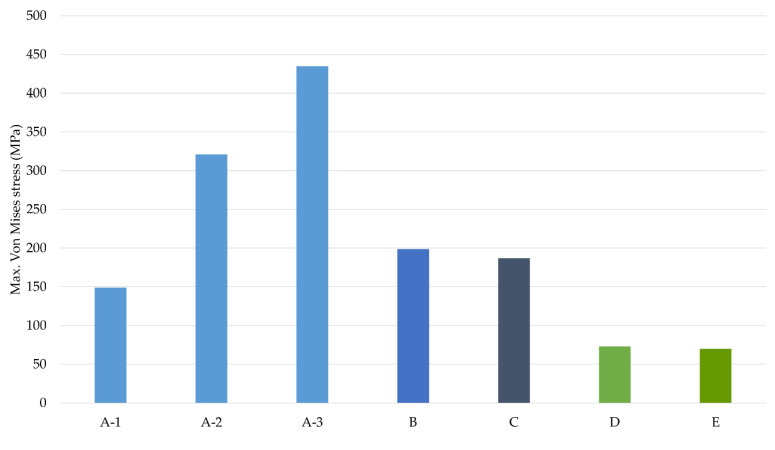
Maximum equivalent von Mises stress values. Blue shades—cases with implants between foramina. Green shades—cases with the 2 implants posterior to the distal of the foramen.

The graph in Figure 5 shows the maximum values of the max. principal strain for each study case (positive values), as well as the minimum values for the min. principal strain (negative values). Scenarios with implants between the foramina are shown in blue shades and scenarios with the two posterior implants positioned distally to the foramina are shown in green shades. The dotted line indicates the level of bone overload that produces a principal strain of 3000 µε, the pathological overload threshold defined by Frost’s bone mechanostat theory [48]. Repeated loads above this threshold produce microdamage to the bone that can overload the bone repair mechanism and lead to fatigue failure [49,50]. The graph in Figure 6 shows the maximum values for the von Mises stresses in the ROIs.

**Figure 7:**
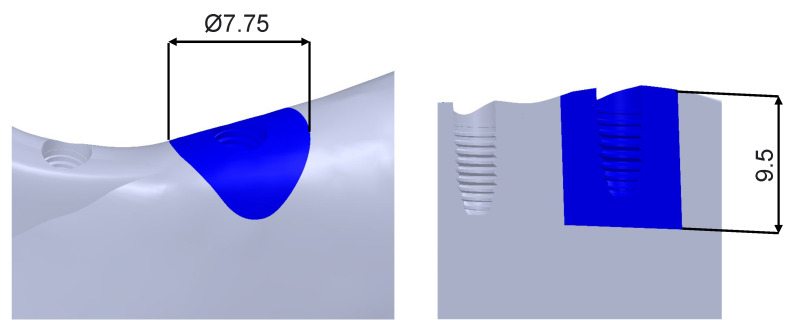
Region of the peri-implant bone in which the bone volume with strain > 3000 µε was calculated.

For each scenario, the peri-implant bone volume with a strain value greater than 3000 µε was calculated. The peri-implant bone volume considered for this calculation corresponds to a cylinder of Ø7.75 mm and a length of 9.5 mm from the implant platform (Figure 7).

**Figure 8:**
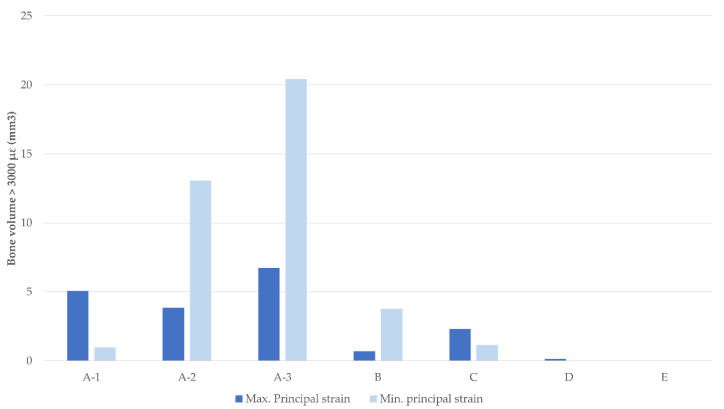
Bone volume with a strain level > 3000 µε for each scenario (mm^3^).

The graph in Figure 8 shows the volumes of bone (mm^3^) with a strain level above 3000 µε calculated for each simulated scenario in the region indicated above.

**Figure 9:**
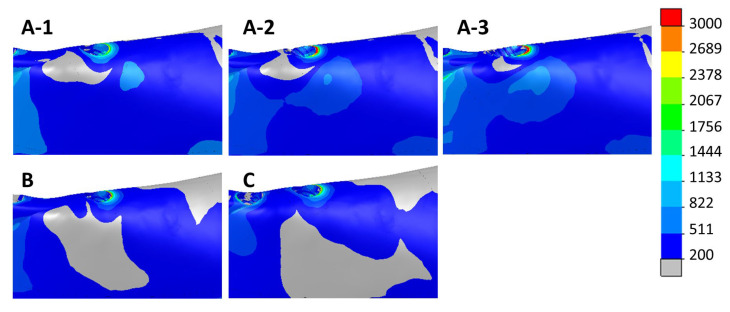
Distribution of the max. principal strain in cases with implants between the foramina. Above—cases with 4 implants (A-1, A-2, and A-3). Below—scenarios with 5 implants (B) and 6 implants (C).

**Figure 10:**
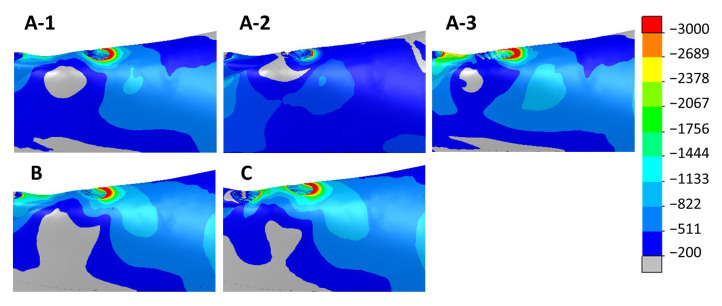
Distribution of the min. principal strain in cases with implants between the foramina. Above—cases with 4 implants (A-1, A-2, and A-3). Below—scenarios with 5 implants (B) and 6 implants (C).

**Figure 11:**
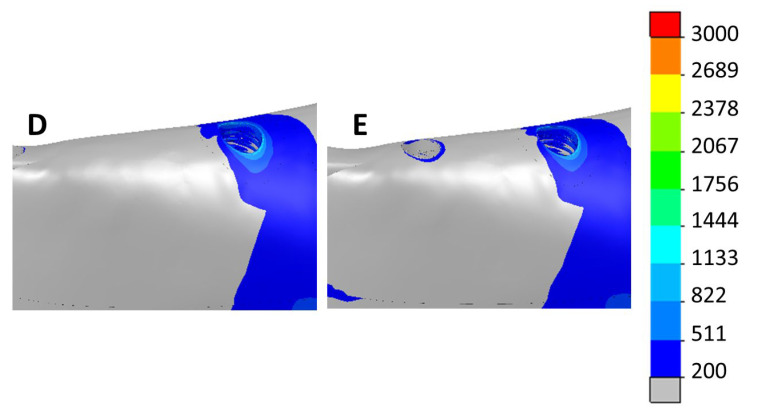
Distribution of the max. principal strain in scenarios with the two posterior implants distally to the foramina. Left—4 implants (D). Right—6 implants (E).

**Figure 12:**
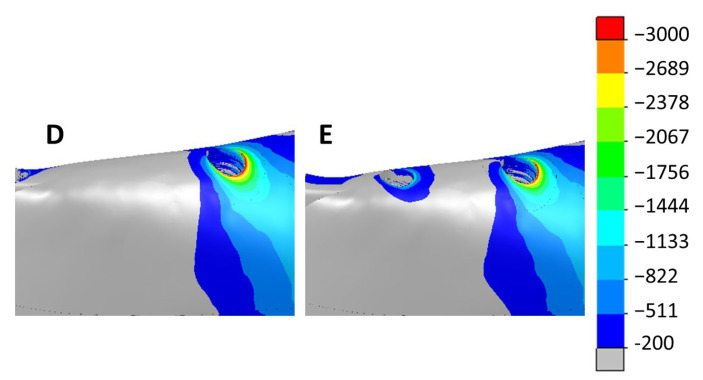
Distribution of the min. principal strain in scenarios with the two posterior implants moved distally to the foramina. Left—4 implants (D). Right—6 implants (E).

Figure 9 and Figure 10 show the max. and min. principal strain distributions in the ROIs for scenarios with implants located between the foramina. Figure 11 and Figure 12 show the max. and min. principal strain distributions in the ROIs for the cases with the two posterior implants located distally to the foramina. Regions with strain values higher than 3000 µε are shown in red.

**Figure 13:**
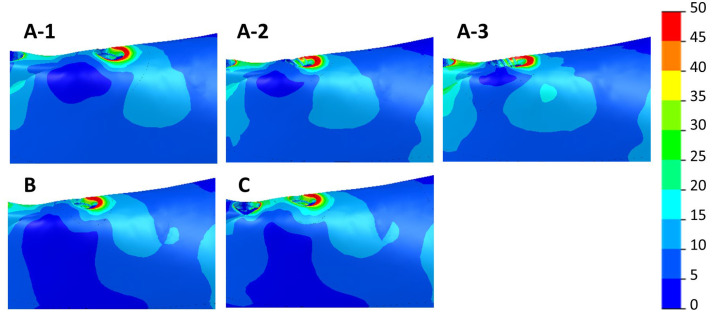
Distribution of the von Mises stresses (MPa) in scenarios A-1, A-2, A-3, B and C.

**Figure 14:**
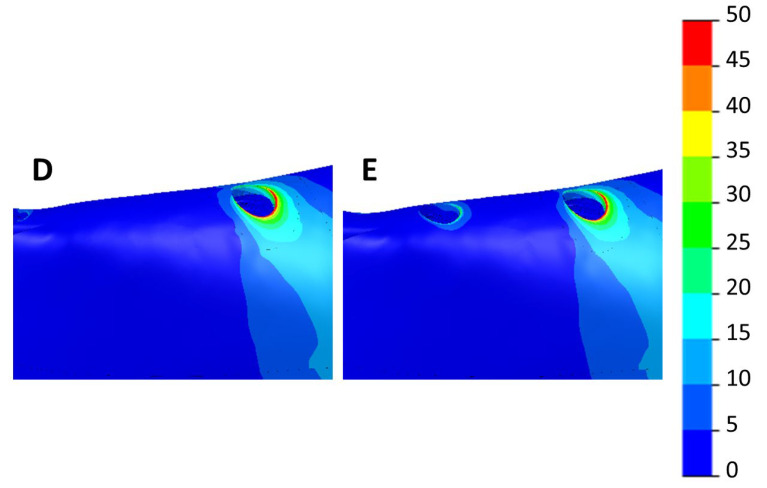
Distribution of the von Mises stresses (MPa) in scenarios with the two posterior implants distally to the foramina. Left—4 implants (D). Right—6 implants (E).

Figure 13 shows the equivalent von Mises stress distributions in the ROIs for scenarios with implants located between the foramina. Figure 14 shows the von Mises stress distributions in the ROIs for those cases with the two posterior implants located distally to the foramina.

## 4. Discussion

From the results obtained, the maximum values of both the strain and stress as well as the highest bone volume with strain values above 3000 µε were observed in cases with four implants, in which the two posterior implants were positioned between the foramina (cases A-1, A-2, and A-3). It is noteworthy that the increase in strains and stresses occurred in the scenarios where posterior implants were angled. In these scenarios, the maximum stress values can be up to three times higher for the case with the posterior implants angled at 45° (Case A-3), compared to the axially placed case (Case A-1). Continuing with the same comparison, i.e., Case A-3 versus Case A-1, principal strains can be up to 2.5 times when tilting the posterior implants. Furthermore, the bone volume with a strain value of more than 3000 µε could be up to 20 times higher. The results obtained in this study agree with those from a FEA performed by Doganay et al., which found that the use of short implants placed distally in such a way that the cantilever was eliminated contributed to the reduction of stresses in the bone surrounding the implants [30]. Similar results were obtained in their study by Bhering et al. using FEA in an edentulous maxilla [51].

Moreover, no cantilever reduction was achieved by implant tilting, with the remaining cases at 11.5 mm. By maintaining the implant emergence point (unaltered and avoiding the dental nerve), the use of prosthetic components that correct the angulation of the implant keeps the fulcrum in the same position.

It can also be observed that clinical scenarios with the two posterior implants placed distally to the foramina, cases D (4 implants) and E (6 implants), showed the lowest strain and stress values of all the clinical situations studied, with the differences between both cases being very little. Moreover, in these two cases, the regions of bones with min. principal strain values above the pathological level (> 3000 µε) were practically zero. In this sense and based on the results obtained in cases with implants placed distally of the foramina, it can be stated that the reduction of distal cantilevers is always a better option from a biomechanical point of view, and the use of two posterior implants placed distally to the foramina represents the best studied alternative.

In patients with edentulous mandibles, the absence of available bone volume in the posterior regions is common and hinders the use of standard-length implants in the axial positions. In such cases (as simulated in scenarios D and E), the use of short or extra-short implants may be helpful and provide a better alternative to vertical bone grafts [3]. This type of implant has already demonstrated good long-term clinical performance in this type of clinical challenge [52,53,54]. The main argument of those studies that supported the use of implants between the foramina by tilting the posterior implants was that it provided a simpler solution to the insufficient bone volume above the dental nerve, providing an acceptable biomechanical solution by reducing the cantilever by angling the posterior implants [4,55]. Although tilting the implants may reduce the cantilever, at the same time, it may result in increased stresses on the peri-implant bone [30,56]. In these clinical situations with distal cantilevers, the posterior implants may be prone to suffering microdamage, further increased due to mandibular flexion. This can lead to a “lever effect” around the midline of the mandible and, therefore, to a significant increase in stresses on the posterior implants [30].

In view of the aforementioned, the hypothesis of the study was accepted as the results indicate that placing short implants distally to the foramina provides better results in terms of stresses and strains by reducing the cantilever while avoiding implant tilting.

However, it is important to note that the use of FE for the analysis of clinical scenarios involves several simplifications, such as assuming bone tissue as isotropic, linear elastic, and homogeneous material, which do not correspond to the properties of real bone tissue, as it has a very complex structure and anisotropic mechanical properties unique to each individual, and varies over time [30]. Therefore, the strain values obtained in the study may differ from those that would occur in a real environment, although the same simplifications in all of the case studies and calculations using the same mandible geometry, implants, and prosthetic components, allow us to perform a reliable comparative analysis among the different cases under study [26].

Moreover, it is relevant to mention that this study did not simulate the dynamic medium of the human mouth. Thus, humidity, chewing, pH, temperature variations, and other bone conditions should be evaluated in further investigations to corroborate the present findings. Regarding the framework material, in this study, only CoCr frameworks were used to avoid adding more variables to the study, and because the effects of using different material frameworks have already been studied by [39,40,42]. Hence, further research is needed to ensure that the results obtained in this research may be extrapolated to other load scenarios, implant distributions, and/or different implant systems.

## 5. Conclusions

Based on the results of this study, it can be stated that:

- From a biomechanical point of view, cases with posterior implants placed distally to the foramen were demonstrated to be the best options to reduce cantilevers.
- The angulation of posterior implants in cases with implants between the foramina does not necessarily reduce the prosthetic cantilever.
- Tilting posterior implants may cause a significant increase in the stresses and strains at the implant–bone interface.
- No significant differences in bone principal strain and stress were observed for scenarios with four or six implants with the two posterior implants located distally to the foramina.
- Tilting posterior implants at 45° demonstrated to be the worst scenario, showing a dramatic increase in stresses and strains.

## Figures and Tables

**Table 1:** Different scenarios, number, location, angulation of implants, and distal cantilever length of the prosthesis.

Scenario	Implants (*n*)	Implant Position	Implant Angulation	Cantilever Length (mm)
A-1	4	2–Lateral incisor	Axial	11.5
		2–Second premolar		
A-2	4	2–Lateral incisor	Axial	11.5
		2–Second premolar	30° distally tilted	
A-3	4	2–Lateral incisor	Axial	11.5
		2–Second premolar	45° distally tilted	
B	5	1–Between central incisors	Axial	11.5
		2–Lateral incisor		
		2–Second premolar		
C	6	2–Central incisor	Axial	11.5
		2–Canine		
		2–Second premolar		
D	4	2–Canine	Axial	0
		2–Second molar		
E	6	2–Lateral incisor	Axial	0
		2–Second premolar		
		2–Second molar		

**Table 3:** Maximum values for the max. principal strain, minimum values for the min. principal strain, and maximum values for the equivalent von Mises stresses for each of the scenarios analyzed.

Scenario	Implant Distribution	Max. Principal Strain (µε)	Min. Principal Strain (µε)	Von Mises Stress (MPa)	Cantilever (mm)
A-1	4 Axial implants	2672	−9362	149	11.5
A-2	4 Implants, 2 tilted at 30°	7151	−20,810	321	11.5
A-3	4 Implants, 2 tilted at 45°	7200	−21,250	435	11.5
B	5 Axial implants	3359	−11,150	199	11.5
C	6 Axial implants	3144	−9477	187	11.5
D	4 Implants: 2 distally placed from the foramina	1669	−4224	72.8	0
E	6 Implants: 2 distally placed from the foramina	1468	−3897	70.1	0

## Data Availability

All the data obtained in this research are described in the manuscript.
